# LY-H as a potential screening biomarker for lymphocyte activation in peripheral blood

**DOI:** 10.3389/fimmu.2026.1767523

**Published:** 2026-03-16

**Authors:** Kang-chun-feng Qin, Chi-hui Yang, Yi-hui Zhang, Xin-yi Wang, Pei-pei Jin, Ning Ding

**Affiliations:** Department of Laboratory Medicine, Ruijin Hospital, Shanghai Jiao Tong University School of Medicine, Shanghai, China

**Keywords:** LY-H, lymphocyte activation, peripheral blood, reactive lymphocytes, viral infections

## Abstract

**Objectives:**

Viral infections often cause morphological changes in peripheral blood lymphocytes. While the presence of reactive lymphocytes in blood smears reflects lymphocyte activation, their identification is often affected by morphological heterogeneity and observer subjectivity. This study evaluates the correlation between LY-H, defined as the vector sum of lymphocyte parameters LY-Y and LY-Z, and the presence of reactive lymphocytes in peripheral blood smears, exploring its potential as a screening marker for lymphocyte activation.

**Methods:**

Data from 404 individuals were collected and divided into four groups: 109 individuals with reactive lymphocytes found in peripheral blood smears (reactive lymphocyte group), 104 lymphoma patients (lymphoma group), 91 patients with acute lymphocytic leukemia (ALL group), and 100 patients with chronic lymphocytic leukemia (CLL group). Differences in LY-H among the four groups were compared. Logistic regression and smooth spline analysis were used to analyze the predictive value and correlation of lymphocyte parameters with reactive lymphocyte proportion. Receiver operating characteristic (ROC) curves were generated to evaluate the predictive value of LY-H for reactive lymphocytes detected by microscopy.

**Results:**

LY-H levels were significantly higher in the reactive lymphocyte group than in the other three groups (*P* < 0.05). A strong correlation was observed between LY-H and the percentage of reactive lymphocytes. Logistic regression analysis indicated that LY-H is an independent factor in the presence of reactive lymphocytes (OR_LY-H_: 1.017, 95% CI: 1.002–1.190). Compared with the lymphoma, ALL, and CLL groups, LY-H demonstrated the highest screening value, with area under the curve (AUC) values of 0.8489, 0.8831, and 0.8943, respectively.

**Conclusions:**

Novel parameter LY-H correlates closely with the reactive lymphocytes identified in peripheral blood smears. It serves as a potential indicator for assessing lymphocyte activation status in clinical practice.

## 1 Introduction

Currently, the detection of reactive lymphocytes in peripheral blood still relies mainly on manual microscopic examination, making identification difficult and increasing the risk of misdiagnosis. Reactive lymphocytes are lymphocytes that have morphological and functional changes in response to antigenic stimulation, such as viral infections, autoimmune diseases, or hematologic malignancies (1, 2). These cells are characterized by morphological features, including increased size, abundant and basophilic cytoplasm—hallmarks of immune system activation. A significant increase in peripheral reactive lymphocytes is frequently observed in several viral diseases, notably infectious mononucleosis (EBV-induced), cytomegalovirus infection, dengue fever, and COVID-19 (1, 3, 4). It is important to recognize that similar morphological abnormalities in lymphocytes may also occur in the early stages of certain hematologic malignancies, thus complicating the diagnosis process (5).

Viruses recognize specific receptors on the surface of lymphocytes via their surface proteins. After invading the cells, they utilize the host’s ribosomes and nucleic acid synthesis systems for massive replication, interfering with the normal material transport and structural maintenance of the cells, which results in increased cell volume and cytoplasmic loosening (6). Additionally, viral-specific CD4+ T cells interact with dendritic cells (DCs) that also engage reactive CD8+ T cells. These CD4+ T cells enhance CD8+ T cell stimulation by promoting costimulatory signaling and viral antigen display on APCs (7). Activated lymphocytes initiate a proliferation program, accompanied by extensive synthesis of intracellular organelles such as ribosomes and mitochondria, leading to a significant increase in cell volume, a change in cytoplasm from basophilic to deep blue, and a decreased nuclear-cytoplasmic ratio. Activated immune cells secrete large amounts of cytokines (IL-2, IFN-γ, TNF-α), which further promote lymphocyte activation and proliferation, and induce cytoskeletal reorganization, transforming the lymphocyte morphology into an “activated phenotype” (8). These morphological alterations represent specific T-cell activation rather than cellular swelling. During an acute viral infection, the host mounts an immune response characterized by the activation of CD8+ effector T-cells, which are morphologically identified as reactive lymphocytes (9). While these morphological shifts are clinically significant, their manual assessment is labor-intensive and subjective. Therefore, exploring the correlation between the LY-H parameter and T-cell subsets (CD3+, CD8+) not only refines the precision of cellular classification but also provides insights into systemic immune status via routine hematological parameters. Meanwhile, excessive pro-inflammatory cytokines cause cellular stress, exacerbating morphological abnormalities.

Although manual microscopy is considered the “reference standard” for detecting reactive lymphocytes, the inherent drawbacks of subjectivity, morphological heterogeneity, and poor reproducibility limit its use in high-throughput clinical laboratories (10, 11). The significant diversity in reactive lymphocytes morphology further complicates visual identification, often leading to classification bias. Therefore, the development of an objective, rapid, and efficient automated method to assist in the screening of reactive lymphocytes is crucial for reducing laboratory workload, improving cell identification efficiency, and ensuring standardized results. The Mindray BC-6800Plus automated hematology analyzer employs a unique SF Cube three-dimensional analytical technology, which integrates three optical signals: forward scatter (FSC) indicates cell size, side scatter (SSC) indicates internal structure and content, and side fluorescence (SFL) indicates the amount of intracellular nucleic acids (12, 13). The analyzer introduces novel lymphocyte parameters: LY-X (cellular complexity, derived from SSC), LY-Y (nucleic acid content, derived from SFL), and LY-Z (cell size, derived from FSC). These parameters offer the potential for an objective and quantitative assessment of morphological changes in lymphocytes, enabling an automated screening of reactive lymphocytes (14, 15). Compared with traditional manual microscopy or costly flow cytometry, this method is simpler to operate, more cost-effective, and better suited for widespread clinical use.

This study aims to systematically evaluate the clinical utility of LY-H (the vector sum of LY-Y and LY-Z) as novel biomarker for detecting reactive lymphocytes in peripheral blood. By comparing the LY-H in patients with reactive lymphocytes and those with other neoplastic lymphocytes, we aim to investigate whether LY-H can provide a more objective and quantitative assessment than traditional morphology. This approach seeks to assist clinicians in the preliminary screening of activated reactive lymphocytes.

## 2 Materials and methods

### 2.1 Study participants

A retrospective analysis was conducted on 404 blood samples collected from outpatient and hematology inpatients at the north campus of Ruijin Hospital, Shanghai Jiao Tong University School of Medicine, between January 2024 and June 2025. The cohort comprised 109 cases with reactive lymphocytes observed in peripheral blood smear (reactive lymphocyte group), 104 cases of pathologically confirmed lymphoma (lymphoma group), 91 cases of acute lymphoblastic leukemia (ALL group), and 100 cases of chronic lymphocytic leukemia (CLL group). Patients were enrolled based on the following criteria (1): reactive lymphocyte group: patients with viral infection via serological or molecular evidence [positive tests for specific IgM antibodies: Epstein-Barr virus (EBV), cytomegalovirus (CMV), respiratory syncytial virus (RSV), adenovirus (ADV), and herpes simplex virus (HSV), or detection of viral DNA/RNA: EBV-DNA or CMV-DNA] and peripheral blood smears confirmed by manual microscopic examination to contain >5% reactive lymphocytes (2). lymphoma group: newly diagnosed patients with Non-Hodgkin Lymphoma (NHL) or Hodgkin Lymphoma (HL) confirmed by histopathology, immunohistochemistry, or flow cytometry (3). ALL and CLL group: newly diagnosed patients meeting the Criteria for Diagnosis and Therapeutic Effect of Hematologic Diseases, confirmed by bone marrow aspiration, cytogenetics, and immunophenotyping. The exclusion criteria were as follows (1): patients who had received chemotherapy, radiotherapy, or immunosuppressive therapy within two weeks before sample collection (2). patients with severe organ failure (acute heart failure or end-stage renal disease) (3). suboptimal specimens, including samples with hemolysis, clots, or a delay in delivery exceeding two hours after collection. To further assess the clinical value of new lymphocyte parameters, the reactive lymphocyte group was subdivided into two subgroups based on manual microscopy results: reactive lymphocyte-positive (≥10%, N = 57) and reactive lymphocyte-negative (<10%, N = 52). This study was conducted in accordance with the Declaration of Helsinki, with ethical approval granted by the Ruijin Hospital Ethics Committee [Ethics No (2019) (54).:], and all participants provided informed consent.

### 2.2 Methods

#### 2.2.1 Sample collection

For each case, 4 mL of fasting venous blood was collected in the early morning, with 2 mL each in EDTA anticoagulant tubes. All samples were tested within two hours of collection.

#### 2.2.2 Laboratory analysis

The novel lymphocyte parameters were obtained using the Mindray BC-6800Plus fully automated hematology analyzer (Mindray, China). Analysis of T-cell subsets (CD3+, CD4+, and CD8+) was conducted with the Mindray BriCyte^®^ MX fully automated flow cytometer, strictly following the manufacturers’ operational protocols. Routine maintenance and quality control were performed for parameter accuracy. Laboratory testing parameters included white blood cell count (WBC), relative lymphocyte count (LY), and the new lymphocyte parameters LY-X, LY-Y, and LY-Z, as well as T-cell subsets (CD3+, CD4+, CD8+). In addition, the calculated parameter LY-H was defined as the vector sum of LY-Y and LY-Z.

### 2.3 Statistical analysis

Statistical analyses were performed using R software and GraphPad Prism 10. For categorical data, the Chen-Shapiro test was applied to assess normality. The normal distribution data were presented as mean ± standard deviation ( $\bar{x}$ ± s), and pairwise comparisons were conducted using the t-test. For non-normally distributed data, results were presented as the median (M) with interquartile range (P25–P75), and pairwise comparisons were conducted using the Mann-Whitney U test. Metric data were expressed as percentages (%), with pairwise comparisons evaluated using the χ² test. LY-H and LY series parameters were standardized through Z-score transformation, followed by smoothing spline analysis, which examined the relationship between LY-H and these parameters and the percentage of reactive lymphocytes. Pearson’s linear correlation analysis was performed to explore associations between LY-H with the LY parameters and T-cell subsets (CD3+, CD4+, CD8+). Logistic regression analysis was carried out to determine the roles of LY-H as risk factors for reactive lymphocytes in peripheral blood smear. Two logistic regression models were constructed: Model 1 was unadjusted, while Model 2 was adjusted for age, sex, WBC, LY#, CD3+, CD4+, and CD8 +. Multivariate logistic regression was conducted to identify the independent effects of LY-H on reactive lymphocytes. The clinical value of LY-X, LY-Y, LY-Z, and LY-H for reactive lymphocytes was further evaluated using receiver operating characteristic (ROC) curves. Statistical significance was defined as *P*< 0.05.

## 3 Results

### 3.1 Demographic data and laboratory results

**Figure 1:**
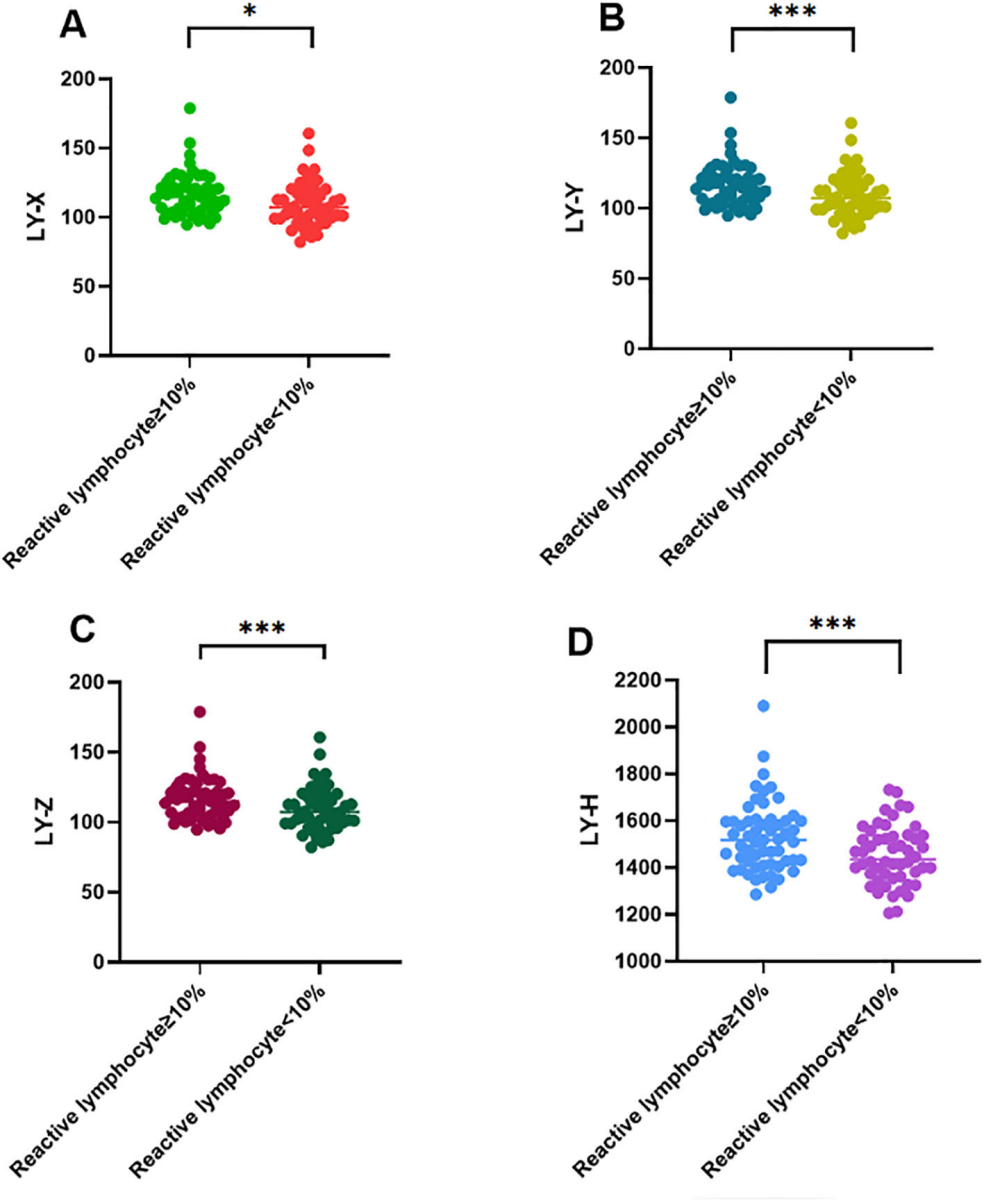
Comparisons of LY-X **(A)**, LY-Y **(B)**, LY-Z **(C)** and LY-H **(D)** levels among reactive lymphocyte ≥10% group and reactive lymphocyte <10% group in patients with reactive lymphocytes in peripheral blood smear. Data are expressed as median and interquartile range. *P < 0.05, ***P < 0.001.

The demographic and laboratory results of all subjects are shown in **Table 1**. No significant differences were observed in sex among the groups. However, there were statistically significant differences in WBC, LY#, LY-Y, LY-Z, and LY-H among the four groups (*P* < 0.05). Furthermore, comparative analysis of lymphocyte parameters suggested that, except for LY-X between the reactive lymphocyte and lymphoma groups, all other parameters showed highly significant differences when comparing the reactive lymphocyte group to each of the disease groups (*P* < 0.01). Additionally, as shown in **Supplementary Table 1** and **Figure 1**, the median values for LY-X, LY-Y, LY-Z, and LY-H were significantly higher in the group with reactive lymphocytes ≥10% compared to the group with <10% (*P* < 0.001).

**Table 1:** Demographic, clinical, and laboratory characteristics among different groups.

		Reactive lymphocyte group	Lymphoma glroup	ALL group	CLL Group	F/χ^2^	*P* value
No. of participants, N (%)		109 (26.98)	104 (25.74)	91 (22.52)	100 (24.75)		
Sex	Male, N (%)	62 (56.88)	65 (62.50)	46 (50.55)	67 (67.00)	6.05	0.109
	Female, N (%)	47 (43.12)	39 (37.50)	45 (49.45)	33 (33.00)		
Age (years), mean ± SD		21.77 ± 20.36	55.11 ± 16.09	56.86 ± 16.46	54.33 ± 9.78	114.3	<0.001
Laboratory results median (25% percentile, 75% percentile)							
WBC (10^9^/L)		8.82 (6.42, 12.47)	4.39 (3.44, 6.41)^a^	4.93 (3.43, 7.45)^b^	15.63 (6.24, 28.22)^c^	37.06	<0.001
LY# (10^9^/L)		5.25 (3.10, 9.28)	0.98 (0.68, 1.58)^a^	1.07 (0.77, 2.07)^b^	12.01 (2.08, 23.65)^c^	39.31	<0.001
LY-X		111.6 (101.6, 121.0)	101.3 (97.7, 105.7)	101.8 (96.9, 105.3)^b^	97.2 (92.5, 105.0)^c^	2.50	>0.05
LY-Y		967.5 (876.2, 1063)	809.4 (775.5, 859.4)^a^	801.7 (765.7, 859.4)^b^	765.4 (715.0, 816.5)^c^	72.51	<0.001
LY-Z		1118 (1077, 1172)	1050 (1030, 1073)^a^	1044 (1026, 1063)^b^	1035 (1011, 1066)^c^	44.91	<0.001
LY-H		1474 (1398, 1581)	1332 (1292, 1365)^a^	1315 (1279, 1352)^b^	1286 (1240, 1348)^c^	73.94	<0.001
CD3+ (%)		77.90 (68.35, 84.20)	79.60 (66.80, 86.00)	81.40 (73.43, 86.60)	74.85 (66.80, 80.18)	2.09	0.102
CD4+ (%)		24.50 (16.40, 31.10)	37.50 (30.30, 46.70)^a^	37.35 (31.45, 46.90)^b^	37.25 (30.75, 44.48)^c^	36.40	<0.001
CD8+ (%)		45.90 (38.10, 58.20)	35.10 (26.50, 43.70)^a^	38.15 (30.48, 45.75)^b^	33.20 (27.43, 37.95)^c^	28.93	<0.001

WBC, white blood count; LY#, lymphocyte count; LY-X, cellular complexity; LY-Y, nucleic acid content; LY-Z, cell size; LY-H, the vector sum of LY-Y and LY-Z; CD3+, CD3+ T cells percentage; CD4+, CD4+ T cells percentage; CD8+, CD8+ T cells percentage; a: indicates comparison with Reactive Lymphocyte Group; b: indicates comparison with Reactive Lymphocyte Group; c: indicates comparison with Reactive Lymphocyte Group; a, b, c: indicates *P* < 0.001.

### 3.2 Logistic regression analysis of novel lymphocyte parameters in predicting reactive lymphocytes

Univariate logistic regression analysis showed that LY-Y, LY-Z, and LY-H were risk factors for reactive lymphocytes in peripheral blood smear (OR_LY-Y_: 1.015, 95% CI: 1.012-1.018, OR_LY-Z_: 1.020, 95% CI: 1.015-1.025, OR_LY-H_: 1.013, 95% CI: 1.011-1.016). Multivariate regression analysis showed that LY-H was an independent risk factor for the occurrence of reactive lymphocytes (OR_LY-H_: 1.017, 95% CI: 1.002-1.190), as shown in **Supplementary Table 2**. To further analyze the predictive value of LY-H for reactive lymphocytes, we divided LY-X, LY-Y, LY-Z, LY-H into quartiles (P25-P75) based on the median (M) and performed multivariable logistic regression. After adjusting for confounding factors (age, sex, WBC, LY#, CD3+, CD4+, and CD8+), LY-H (1341.12 < LY-H ≤ 1410.01 and LY-H > 1410.01) was independently associated with reactive lymphocytes in peripheral blood smear, with LY-H ≤1287.34 as the reference group (Q1), as shown in **Table 2**.

**Table 2:** Individual effect of lymphocyte parameters on predicting reactive lymphocytes in peripheral blood smear.

	Adjusted model I		Adjusted model II	
	OR (95% CI)	*P* value	OR (95% CI)	*P* value
LY-H groups				
Q1 (LY-H ≤ 1287.3)	Reference		Reference	
Q2 (1287.3 < LY-H ≤ 1341.1)	1.21 (0.36, 4.11)	0.76	0.99 (0.28, 3.46)	0.99
Q3 (1341.1 < LY-H ≤ 1410.0)	5.04 (1.82, 13.97)	<0.01	3.45 (1.09, 10.90)	0.04
Q4 (LY-H > 1410.0)	61.60 (22.46, 168.97)	<0.01	24.49 (4.86, 123.47)	<0.01
LY-X groups				
Q1 (LY-X ≤ 96.8)	Reference		Reference	
Q2 (96.8 < LY-X ≤ 102.1)	1.18 (0.53, 2.63)	0.68	0.24 (0.08, 0.69)	<0.01
Q3 (102.1 < LY-X ≤ 108.8)	1.61 (0.75, 3.45)	0.23	0.11 (0.03, 0.37)	<0.01
Q4 (LY-X > 108.8)	10.21 (5.04, 20.68)	<0.01	0.03 (0.01, 0.14)	<0.01
LY-Y groups				
Q1 (LY-Y ≤ 766.7)	Reference		Reference	
Q2 (766.7 < LY-Y ≤ 823.2)	1.23 (0.36, 4.15)	0.74	0.83 (0.22, 3.17)	0.79
Q3 (823.2 < LY-Y ≤ 897.8)	5.66 (2.06, 15.58)	<0.01	1.77 (0.43, 7.31)	0.43
Q4 (LY-Y > 897.8)	53.33 (19.60, 145.10)	<0.01	4.08 (0.52, 31.92)	0.18
LY-Z groups				
Q1 (LY-Z ≤ 1031.3)	Reference		Reference	
Q2 (1031.3 < LY-Z ≤ 1054.4)	1.16 (0.40, 3.31)	0.79	1.34 (0.38, 4.70)	0.65
Q3 (1054.4 < LY-Z ≤ 1095.8)	3.74 (1.52, 9.21)	<0.01	4.05 (1.12, 14.62)	0.03
Q4 (LY-Z > 1095.8)	33.34 (13.82, 80.43)	<0.01	19.78 (3.26, 119.85)	<0.01

Adjusted Model I: Non-adjusted.

Adjusted Model II: Adjusted for age, sex, WBC, LY#, CD3+, CD4+, and CD8+.

### 3.3 Smooth spline analysis of new lymphocyte parameters and reactive lymphocyte proportion

**Figure 2:**
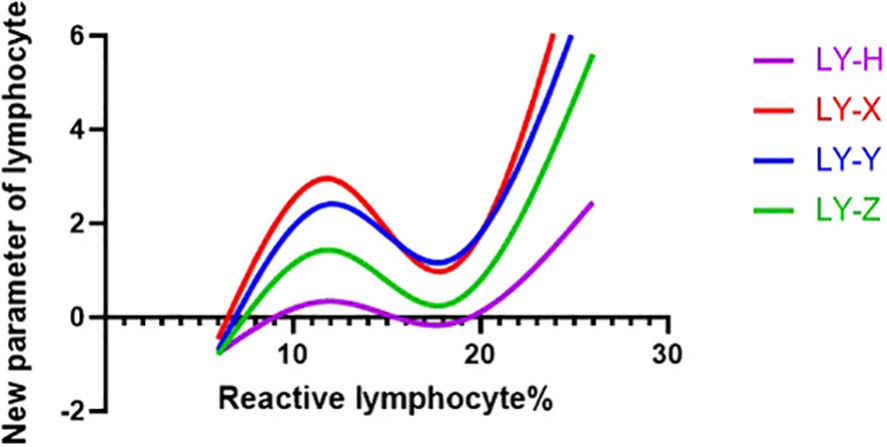
Smoothing spline analysis of LY-X, LY-Y, LY-Z and LY-H and percentage of reactive lymphocytes.

As the proportion of reactive lymphocytes increased, LY−X, LY−Y, and LY−Z rose initially, declined, and then rose again. In contrast, LY−H followed a steady upward trend, showing only minor fluctuations before continuing its marked ascent (**Figure 2**). LY−H maintained a steady relationship with reactive lymphocyte proportion, indicating its reliability as an indicator.

### 3.4 Correlation between LY-H with LY-X, LY-Y, LY-Z, WBC, LY#, CD3+, CD4+ and CD8+ in the reactive lymphocytes group

**Figure 3:**
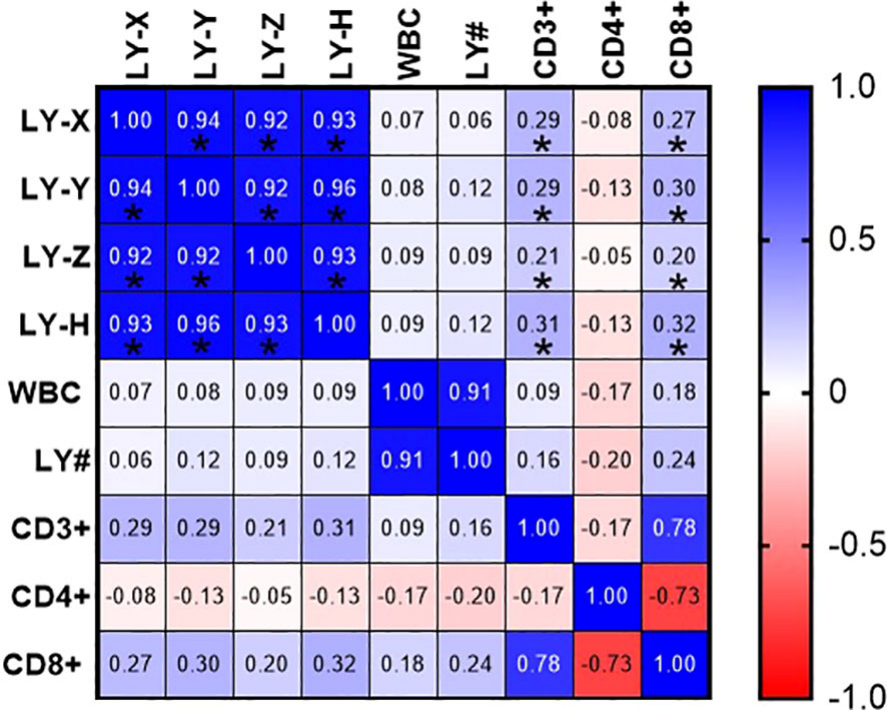
Correlation between LY-X, LY-Y, LY-Z and LY-H with WBC, LY#, CD3+, CD4+ and CD8+. *P < 0.05.

Pearson linear correlation analysis showed that LY-H were positively correlated with LY-X, LY-Y, LY-Z, CD3+ and CD8+, with correlation coefficients of 0.93, 0.96, 0.93, 0.31, 0.32. All differences were statistically significant (*P*<0.05), as presented in **Figure 3**.

### 3.5 ROC curves of novel lymphocyte parameters for predicting reactive lymphocytes in peripheral blood smear

**Figure 4:**
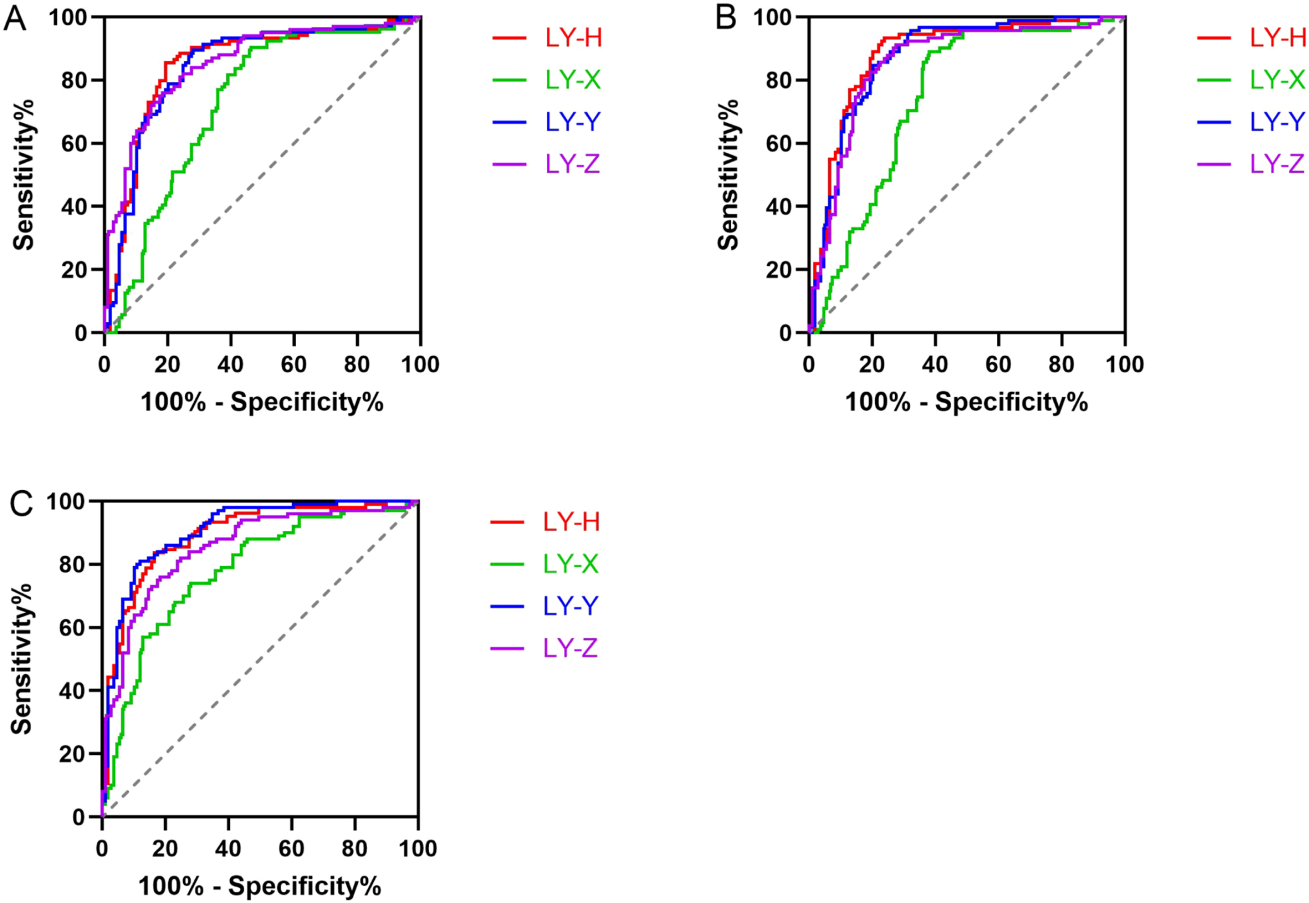
ROC curves of LY-X, LY-Y, LY-Z and LY-H for predicting reactive lymphocyte activation in different disease groups. **(A)** Reactive Lymphocyte Group vs. Lymphoma Group. **(B)** Reactive Lymphocyte Group vs. ALL Group. **(C)** Reactive Lymphocyte Group vs. CLL Group.

Across all comparison groups, LY-H demonstrated the best screening performance, with its area under the curve (AUC) showing an advantage in different disease categories. Specifically, when compared to the lymphoma and ALL groups, LY-H yielded the highest AUC values at 0.8489 (cutoff: 1382; sensitivity: 85.58%; specificity: 80.73%) and 0.8831 (cutoff: 1373; sensitivity: 82.42%; specificity: 81.65%), respectively. In the comparison with the CLL group, the AUC for LY-H was 0.8943 (cutoff: 1371; sensitivity: 83.65%; specificity: 82.57%), which was slightly lower than that of LY-Y (AUC: 0.9079). In addition to LY-H, both LY-Y and LY-Z also exhibited strong predictive value, with their AUCs ranging between 0.84 and 0.91, as detailed in **Supplementary Table 3** and **Figure 4**.

## 4 Discussion

With the continuous advancement of hematology analyzers, an increasing number of blood cell parameters can sensitively and accurately reflect changes in cell morphology, size, and nucleic acid content, providing valuable references for disease screening, diagnosis, and treatment (16). For example, neutrophil parameters have diagnostic utility in diseases such as sepsis, chronic myelomonocytic leukemia, and myelodysplastic syndromes (17–19). However, lymphocyte parameters are influenced by multiple factors, including viral infections, and their role in differentiating benign from malignant lymphocytes or among different lymphomas remains unclear (20). Chhuy et al. (21) effectively detected mantle cell lymphoma (MCL) by combining LY−X with instrument−generated abnormal lymphocyte alerts, even in cases without elevated absolute lymphocyte counts or abnormalities in hemoglobin and platelet levels. Furundarena et al. (20) conducted a comprehensive analysis of LY−X, LY−Y, LY−Z, LY−WX, LY−WY, and LY−WZ across various lymphomas, and successfully identified cases of large granular T−cell lymphoma using these parameters. In our study, LY-H demonstrated superior screening performance in predicting peripheral blood lymphocyte activation across all disease categories, with its AUC showing a distinct advantage in each comparative analysis. As a novel biomarker, LY-H is simple, non-invasive, and characterized by high sensitivity and specificity, offering significant value for the effective screening of reactive lymphocyte activation.

Our findings reveal that LY-Y (nucleic acid content), LY-Z (cell size), and the novel parameter LY-H are significantly elevated in the reactive lymphocyte group compared to the malignant lymphoid tumor groups. This suggests that these parameters effectively capture the unique activation state of reactive lymphocytes, which differs from the proliferation patterns observed in many neoplastic diseases. Reactive lymphocytes typically exhibit increased cellular volume (increased LY-Z) and higher nucleic acid content (elevated LY-Y). These changes can be attributed to the heightened metabolic activity of proteins and nucleic acids during an immune response, such as those triggered by viral infections (22, 23). This further indicates that these parameters are highly sensitive to lymphocyte activation specifically, rather than being a mere reflection of general cell proliferation. Consequently, our results demonstrated LY-Y, LY-Z, and LY-H as reliable potential indicators for the early screening of lymphocyte activation.

Notably, our smoothing spline analysis revealed a significant non-linear relationship between the parameters (LY-X, LY-Y, and LY-Z) and the percentage of reactive lymphocytes. Specifically, these parameters reached a peak when the reactive lymphocyte proportion was near 10%, dropped to their lowest point at approximately 20%, and then began to rise again. This three-phase fluctuation is interesting; however, since this is a cross-sectional study, we lack longitudinal follow-up data. Therefore, this pattern likely reflects the differences in cell populations among patients at various stages or severity levels of the disease. From a morphological perspective, reactive lymphocytes typically show increased cell volume and higher nucleic acid content in the cytoplasm following antigen stimulation (24). Based on current immunology, we propose the following interpretations for this three-phase trend. Initial rise: the synchronized increase in these parameters may correspond to the early stage of immune activation, where many lymphocytes transform into an active state (25). Intermediate decline: the exact reason for the drop at middle proportions is not yet clear. It might be related to the start of immune feedback or a shift in cell subsets (such as regulatory cells), which temporarily lower the average morphological values (26, 27). Secondary rise: the later increase at higher proportions likely reflects the further expansion of highly active effector cells under continuous stimulation (28). However, these points are still experimental hypotheses. The direct link between these parameters and immune function needs to be confirmed by future studies using long-term monitoring and functional markers like cytokines. In contrast, the composite parameter LY-H (the combination of LY-Y and LY-Z) showed a much more stable and consistent upward trend. Biologically, when CD8+ T cells are activated into effector cells, their internal structures and size increase significantly. Because LY-H combines both nucleic acid intensity (LY-Y) and cell volume (LY-Z), it can capture the physical changes of activated lymphocytes more effectively than any single parameter. Our finding that LY-H correlates positively with CD3+ and CD8+ T cell counts further supports its value as a potential marker for assessing immune status, though this needs to be verified in larger clinical trials.

Additionally, apart from LY-X, LY-Y, and LY-Z, the Mindray BC-6800Plus analyzer offers the “High Fluorescent Cell (HFC)” parameter, which helps to identify immature cells and reactive lymphocytes. Previous studies have shown that the HFC parameter provides high sensitivity in detecting viral infections such as dengue fever (29, 30). Although this study did not explore an in-depth analysis of HFC, the detailed information provided by LY-Y and LY-Z regarding cellular activation suggests that combining these parameters with HFC and other markers could help establish more complete and reliable screening tools.

Reactive lymphocytes are most frequently observed in patients’ peripheral blood due to viral infections, which primarily target B lymphocytes. The human body responds by launching a strong cellular immune reaction, leading to an activation of effector T cells and a relative suppression of regulatory T cells (31). This process, particularly of CD8+ T cells, results in elevated CD3+ levels and indicates effective cellular immune activation. In cases of EBV infection, B cells present viral antigens that activate and drive the proliferation of CD8+ T cells, resulting in the formation of “activated cytotoxic T cells,” which morphologically recognized as “reactive lymphocytes” (32). EBV may also indirectly inhibit the proliferation of CD4+ T cells, prompting the immune system to preferentially mobilize CD8+ T cells for cytotoxic activity, thereby reducing the relative proportion of CD4+ T cells among the total T cell population. In this study, we first observed that LY-Y, LY-Z, and LY-H correlate positively with CD3+ and CD8+ T cell counts, suggesting their potential as biomarkers for assessing immune status. Reactive lymphocytes primarily consist of activated CD8+ T cells. When naive CD8+ T cells bind to MHC-I molecules, they are activated into effector cytotoxic T lymphocytes (CTLs), which secrete cytotoxic granules containing perforin and granzymes (33). This biological process concurrently leads to an increase in both nucleic acid fluorescence intensity (LY-Y) and cell volume (LY-Z). As a composite vector of LY-Y and LY-Z, LY-H reflects the activated lymphocyte phenotype more comprehensively and with greater sensitivity than any single parameter. Furthermore, lymphocyte activation triggers cell proliferation and an increase in nucleic acid content (DNA and RNA) to support the synthesis of additional proteins and organelles required for the immune response (34, 35). Particularly in benign reactive conditions triggered by viral infections, lymphocyte activation is highly metabolism-dependent, leading to a marked elevation in LY-H values. In contrast, in hematological malignancies such as ALL, CLL, and lymphoma, the cells undergo monoclonal expansion, which typically lacks complex immune activation and organelle rearrangement (36). Consequently, the increase in LY-H is significantly less pronounced in these neoplastic cells. These observations suggest a potential screening hypothesis: “high LY-H accompanied by elevated CD8+ counts” likely reflects a viral immune response, whereas “low LY-H accompanied by an increase in total lymphocyte count” is more indicative of neoplastic proliferation.

This study is a single-center retrospective analysis with a relatively limited sample size, which has certain limitations. To further validate the effectiveness and generalizability of the LY-Y and LY-Z parameters, future research should conduct multicenter and prospective clinical studies with larger sample sizes. In addition, although this study found correlations between LY-H and the CD3+, CD8+ T cell subsets, it did not explore their specific clinical significance in different disease states in depth. Future research could further investigate the distinct manifestations of these parameters in various pathogen infections (such as CMV and COVID-19) and different types of lymphatic system diseases, enhancing their value in supporting differential diagnosis. Finally, it would be valuable to explore integrating the new LY series parameters with other immunological indicators such as T cell subsets and the neutrophil-to-lymphocyte ratio (NLR), to establish a more comprehensive model for disease screening and immune status evaluation.

## 5 Conclusion

In summary, this study demonstrates that LY-H levels are highly correlated with the activation and morphological changes of peripheral blood lymphocytes, serving as a sensitive biomarker for monitoring cellular immune status. By accurately capturing the combined shifts in nucleic acid content and cell volume during immune activation, LY-H provides an objective reflection of the increased metabolic activity under antigenic stimulation. Furthermore, it enables a rapid clinical assessment of immune response intensity, offering objective and quantitative evidence as supportive data for the diagnosis of related diseases.

## Data Availability

The original contributions presented in the study are included in the article/**Supplementary Material**. Further inquiries can be directed to the corresponding authors.
