# Supplementary material for: LY-H as a potential screening biomarker for lymphocyte activation in peripheral blood
Source: Front Immunol. 2026 Mar 16;17:1767523. doi: 10.3389/fimmu.2026.1767523 (PMC13033647; doi:10.3389/fimmu.2026.1767523)
Supplement: Supplementary file 1 [file Table1.pdf]

Supplementary Tables

Table S1. Comparison of the median levels of LY-X, LY-Y, LY-Z, LY-H in different reactivity lymphocyte percentage groups

Indicators	Reactivity lymphocyte ≥ 10%	Reactivity lymphocyte < 10%	*t*	*P* value
LY-X	116.4 ± 15.17	109.2 ± 15.71	2.413	0.018
LY-Y	1013 ± 134.7	936.4 ± 127.6	3.052	0.003
LY-Z	1146 ± 83.01	1110 ± 58.08	2.681	0.009
LY-H	1531 ± 148.4	1453 ± 124.7	2.955	0.004
WBC (10^9/L)	9.88 ± 4.72	9.71 ± 5.76	0.168	0.867
LY# (10^9/L)	6.98 ± 4.07	5.90 ± 4.28	1.350	0.180
CD3+ (%)	77.75 ± 10.73	74.52 ± 12.63	1.429	0.156
CD4+ (%)	25.19 ± 11.54	24.54 ± 9.96	0.317	0.752
CD8+ (%)	50.76 ± 16.81	47.67 ± 16.82	0.961	0.339

WBC: white blood count; LY#: lymphocyte count; LY-X: cellular complexity; LY-Y: nucleic acid content; LY-Z: cell size; LY-H: the vector sum of LY-Y and LY-Z; CD3+: CD3+ T cells percentage; CD4+: CD4+ T cells percentage; CD8+: CD8+ T cells percentage.

Table S2. Univariate and Multivariate analysis of laboratory indicators and demographic characteristics of the study participants

	Univariate logistic regression analysis			Multivariate logistic regression analysis		
Indicators	OR	95% CI	*P* value	OR	95% CI	*P* value
LY-X	1.007	0.994 - 1.020	0.308	0.719	0.636 - 0.795	<0.001
LY-Y	1.015	1.012 - 1.018	<0.001	1.042	0.985 - 1.060	0.104
LY-Z	1.020	1.015 - 1.025	<0.001	1.009	0.939 - 1.026	0.567
LY-H	1.013	1.011 - 1.016	<0.001	1.017	1.002 - 1.190	0.024
WBC (10^9/L)	0.989	0.972 - 1.007	0.197	0.986	0.850 - 1.134	0.843
LY# (10^9/L)	0.994	0.975 - 1.010	0.458	1.044	0.900 - 1.220	0.562
CD3+ (%)	1.001	0.981 - 1.021	0.950	0.799	0.662 - 0.942	0.006
CD4+ (%)	0.887	0.861 - 0.911	<0.001	1.078	0.918 - 1.285	0.372
CD8+ (%)	1.085	1.063 - 1.110	<0.001	1.229	1.043 - 1.483	0.012

WBC: white blood count; LY#: lymphocyte count; LY-X: cellular complexity; LY-Y: nucleic acid content; LY-Z: cell size; LY-H: the vector sum of LY-Y and LY-Z; CD3+: CD3+ T cells percentage; CD4+: CD4+ T cells percentage; CD8+: CD8+ T cells percentage.

Table S3. Sensitivity, specificity, cut-off value, and ROC curve analysis results of predicting reactive lymphocytes activation in peripheral blood smear for lymphocyte parameters

Indicators	AUC	Sensitivity	Specificity	Cut-off Value	95%CI
Reactive Lymphocyte Group vs. Lymphoma Group					
LY-X	0.7296*	70.19%	66.06%	104.8	0.6573 - 0.7965
LY-Y	0.8439*	76.92%	79.82%	862.7	0.7882 - 0.8997
LY-Z	0.8438*	77.00%	78.90%	1070	0.8015 - 0.9061
LY-H	0.8489*	85.58%	80.73%	1382	0.8031 - 0.9147
Reactive Lymphocyte Group vs. ALL Group					
LY-X	0.7443	70.33%	68.81%	103.8	0.6745 - 0.8140
LY-Y	0.8736*	79.12%	80.73%	861.9	0.8238 - 0.9235
LY-Z	0.8563	81.32%	79.82%	1069	0.8012 - 0.9113
LY-H	0.8831*	82.42%	81.65%	1373	0.8337 - 0.9324
Reactive Lymphocyte Group vs. CLL Group					
LY-X	0.7801*	74,00%	71.56%	103.2	0.7170 - 0.8432
LY-Y	0.9079*	86.00%	79.82%	867.5	0.8678 – 0.9480
LY-Z	0.8535*	78.00%	77.98%	1071	0.8015 – 0.9061
LY-H	0.8943*	83.65%	82.57%	1371	0.8509 – 0.9378

LY-X: cellular complexity; LY-Y: nucleic acid content; LY-Z: cell size; LY-H: the vector sum of LY-Y and LY-Z. *: $P< 0.05$
